# Accuracy of computer-aided geometric three-dimensional reconstruction of the human petrous bone based on serial unstained celloidin sections

**DOI:** 10.3892/etm.2015.2226

**Published:** 2015-01-28

**Authors:** XIAN-FENG WEI, XIAO-YANG ZHANG, WU YUAN, YUN-SHENG LI

**Affiliations:** 1 Department of Anatomy, Histology and Embryology, Basic Medical College, Tianjin Medical University, Tianjin 300070, P.R. China; 2 Department of Otolaryngology Head and Neck, Tianjin First Central Hospital, Tianjin 300192, P.R. China

**Keywords:** three-dimensional reconstruction, petrous bone, serial unstained celloidin sections, spatial relationships

## Abstract

The aim of this study was to present a comprehensive three-dimensional (3D) morphology of the petrous bone with computer image-processing technology, which could be beneficial for the teaching of anatomy and for surgical procedures. The unstained celloidin sections of human temporal bone were digitized with high resolution and quality, and then processed with Amira^®^ software to include alignment, segmentation and reconstruction. The integral structure of the human inner ear was presented with computer modeling, including the petrous bone, bone labyrinth, internal carotid artery canal, internal jugular vein canal, sigmoid sinus, inferior petrosal sinus, glossopharyngeal meatus, vagal meatus, internal acoustic meatus, facial nerve canal, greater superficial petrosal nerve, vestibular aqueduct, extraosseous portion of the endolymphatic sac, round and oval window, processus cochleariformis and pyramidal eminence. The 3D model showed detailed structure of the external and internal petrous bone, as well as their spatial relationship. The present study suggests the feasibility of comprehensive 3D reconstruction of the petrous bone using unstained celloidin sections, which may provide advantages for future study.

## Introduction

The morphological study of the petrous bone is a significant challenge due the complex structure of the bone and its inaccessibility during surgery, as well as the difficulty in preparing qualified specimens. For an inexperienced surgeon, it is necessary to practice with dissections and specimens, as well as to learn from papers and atlases, which presents considerable difficulties. As computing power has developed, computer technology has played an increasingly important role in a variety of research areas; for example, with the most advanced computer technology, it would be possible to generate a virtual model of a dinosaur appearing identical to a real dinosaur (1). The present is thus an appropriate time for conventional methods of surgical training and anatomical learning to be changed accordingly.

Numerous studies (2–4) have reported the use of computer-aided three-dimensional (3D) reconstruction based on serial stained celloidin sections, such as for the morphological study of the inner ear; however, due to the limitations of hardware and software, only parts of sections have been employed, with the sampling of every fifth to 10th section. It is still not possible to generate a 3D reconstruction of the petrous bone with high-resolution computed tomography (CT) (5) or magnetic resonance imaging (MRI) (6). Although micro-CT (7,8) and magnetic resonance microscopy (MRM) (9) have already been used for the 3D reconstruction of the mammalian inner ear, the resolution is too low to inspect detailed structures with precision.

For studies on the petrous bone, the majority of previous reports have focused on a specific part or local structure. There is thus a requirement for a comprehensive and qualified 3D model for the integral structure of petrous bone to be made for the convenience of further study of the relationship between the external and internal portions. On this basis, the aim of the present study was to use serial unstained celloidin sections to reconstruct a comprehensive and qualified 3D model of the petrous bone, with a special focus on the complicated and porous structure of the skull base.

## Materials and methods

### Celloidin sections without staining

The present study utilized cadavers from the Department of Anatomy, Histology and Embryology of Tianjin Medical University (Tianjin, China). Signed informed consent had been provided for the donation of the cadavers for research purposes, and the study was approved by the Ethics Committee of Tianjin Medical University.

The experimental material was from eight adult cadavers fixed with formalin (four males and four females) with an age range of between 56 and 81 years (mean age, 69.4 years). The temporal bones were removed according to the general standards. The specimens were fixed in formalin, decalcified in chlorhydric acid (Tianjin Ting Da Xi Gui Chemical Reagent Factory, Tianjin, China) and embedded in celloidin. Following being hardened in ethanol, the celloidin blocks (Tianjin Da Mao Chemical Reagent Factory, Tianjin, China) were trimmed to the shape of standard cuboids and mounted on a plastic block provided with a sliding microtome (American Optical Sliding Microtome 860; Reichert Technologies, Depew, NY, USA). The petrous bones were serially sectioned in the horizontal plane at a thickness of 40 μm and preserved in 60% ethanol. A right petrous bone was randomly selected for digitization and reconstruction. The right petrous bone originated from a 75-year-old male with no history of otological disease. A total of 487 successive celloidin sections of the petrous bone without staining were finally obtained for the computer demonstration.

### Digitization of unstained sections

Each unstained section was spread on a glass slide measuring 45×60 mm, immersed in 60% ethanol and finally covered by another thin slide of the same size. A digital camera (DC) with a 12-megapixel charge-coupled device (Canon PowerShot A2100 IS; Canon Inc., Tokyo, Japan) was used to capture the images. The focus of the DC, as well as the locations of the DC and the section, were adjusted to ensure that the entire images were exactly covered by the viewfinder. The setup then remained the same for the rest of the procedure. The highest resolution and best definition of the DC were used to generate the images, which were then saved in the digital JPEG picture format. The refined structure was observed through anatomic microscopy (Olympus SZX7 Zoom Stereo Microscope; Olympus Corp., Tokyo, Japan). When the rupture of a section was encountered, the next intact section took its place.

### Reconstruction

The subsequent 3D reconstruction procedure consisted of several steps, which were all accomplished with Amira^®^ software (version 5.4.1; Template Graphics Software, Inc., San Diego, CA, USA) on a high-capability computer (Hasee X86; Shenzhen Hasee Computer Co., Ltd., Beijing, China). The first step was image alignment. As the available sections had no previous embedded fixed markers, the images of the sections were aligned manually following digitization by comparing, translating and rotating adjacent slides with respect to one another and by superimposing one over the next in Adobe Photoshop (version 6.0; Adobe Systems, Inc., San Jose, CA). The frame of reference for the alignment was the outline border of each slide and the regular structures of the petrous bone.

**Figure 1:**
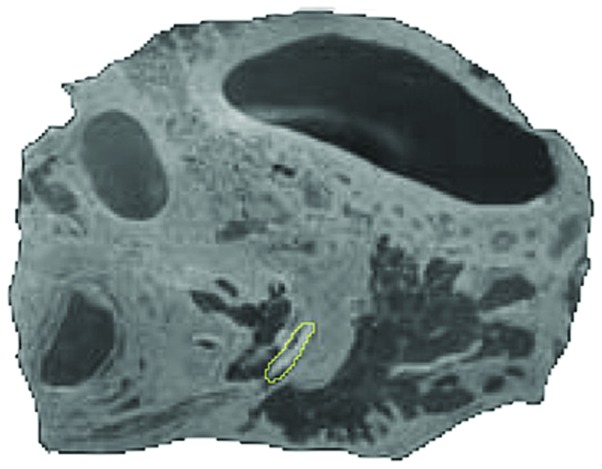
Segmenting the facial nerve canal using the ‘Brush’ tool in Amira^®^ (Template Graphics Software, Inc., San Diego, CA, USA). The yellow line indicates the outline of the facial nerve canal.

The second vital step was segmentation. Once the digitized sections were imported into Amira, the anatomical structures of interest were extracted using the software segmentation tools. The structures of interest included the bone labyrinth, internal carotid artery (ICA) canal, internal jugular vein (IJV) canal, sigmoid sinus (SS), inferior petrosal sinus (IPS), glossopharyngeal meatus, vagal meatus, internal acoustic meatus (IAM), facial nerve canal, greater superficial petrosal nerve, vestibular aqueduct (VA), extraosseous portion of the endolymphatic sac (ES), round and oval window, processus cochleariformis and pyramidal eminence. When the names and colors of the objects were set, the components of the petrous bone were segmented (Fig. 1). The majority of the segmentation work was performed manually by combining the ‘Blow tool’ and ‘Brush’ functions.

The final procedure of the 3D reconstruction followed the segmentation step. Polygonal 3D surface models of the segmented structures were generated, and the surface simplification algorithm of the Amira software was applied to smooth the model by eliminating redundant vertices and reducing the number of triangles in each model. The 3D models were shown in Amira as shaded smooth surfaces with control over rotation and transparency.

## Results

### Establishment of petrous bone 3D model

**Figure 2:**
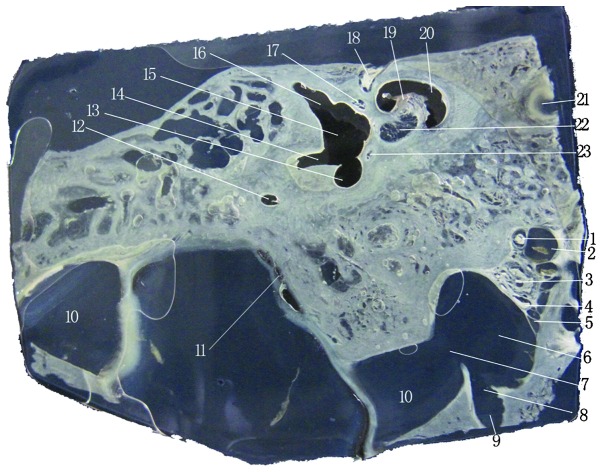
Image of an unstained celloidin section. 1, glossopharyngeal nerve; 2, IPS; 3, vagal nerve; 4, accessory nerve; 5, branch of the IPS draining into the IJV; 6, IJV; 7, SS draining into the IJV; 8, junction of the condylar canal and the IJV; 9, condylar canal; 10, SS; 11, extraosseous portion of endolymphatic sac; 12, posterior semicircular canal; 13, common crus; 14, opening of the crus simplex; 15, vestibule; 16, ampullated end of the superior semicircular canal, which corresponds with the vestibule; 17, superior vestibular nerve; 18, labyrinth part of the facial nerve; 19, modiolus; 20, cochlea; 21, internal carotid artery; 22, cochlear nerve and inferior vestibular nerve; 23, posterior ampullar nerve. IJV, internal jugular vein; SS, sigmoid sinus; IPS, inferior petrosal sinus.

**Figure 3:**
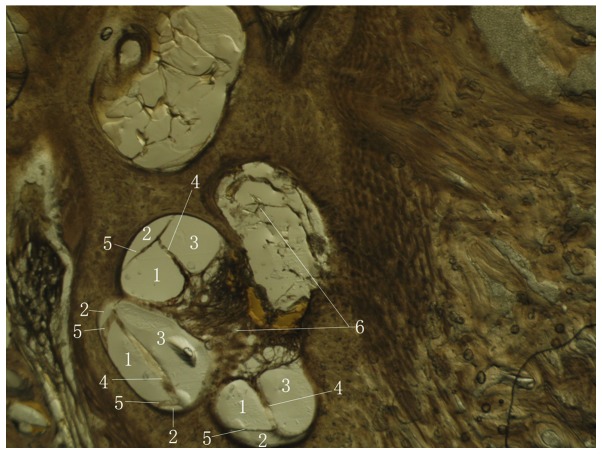
Enlarged structure of the cochlea slice (x6) under observation with anatomic microscopy. 1, scala vestibule; 2, scala media; 3, scala tympani; 4, basilar membrane; 5, Reissner’s membrane; 6, cochlear nerve.

A comprehensive 3D model of integral petrous bone was established on the basis of a complete set images of the serial sections. As shown in Fig. 2, the contour of the detailed structure was clearly imaged with high precision, including the posterior ampullar nerve, the branch of the IPS and the ES. It was possible to display different parts of the structure in different ways using outlining, shading, lining, pointing, transparency and contrasting patterns. It was also possible to determine which parts would be displayed or hidden, which could be useful in providing insights into the complex structure of human petrous bone. Of note, the structure of the locations labeled in Fig. 2 (such as locations 1–5, 11, 17, 22 and 23) can only be observed in celloidin slices, rather than through the use of CT and MRI, which strongly confirms the advantages of our model compared with conventional imaging methods. Under anatomic microscopy, the details of the refined structure could be observed clearly in an enlarged view. As shown in Fig. 3, enlargement of the components inside of the cochlea revealed the two partitive membranes and three lumen.

### Association between VA, ES and the bony labrynth

**Figure 4:**
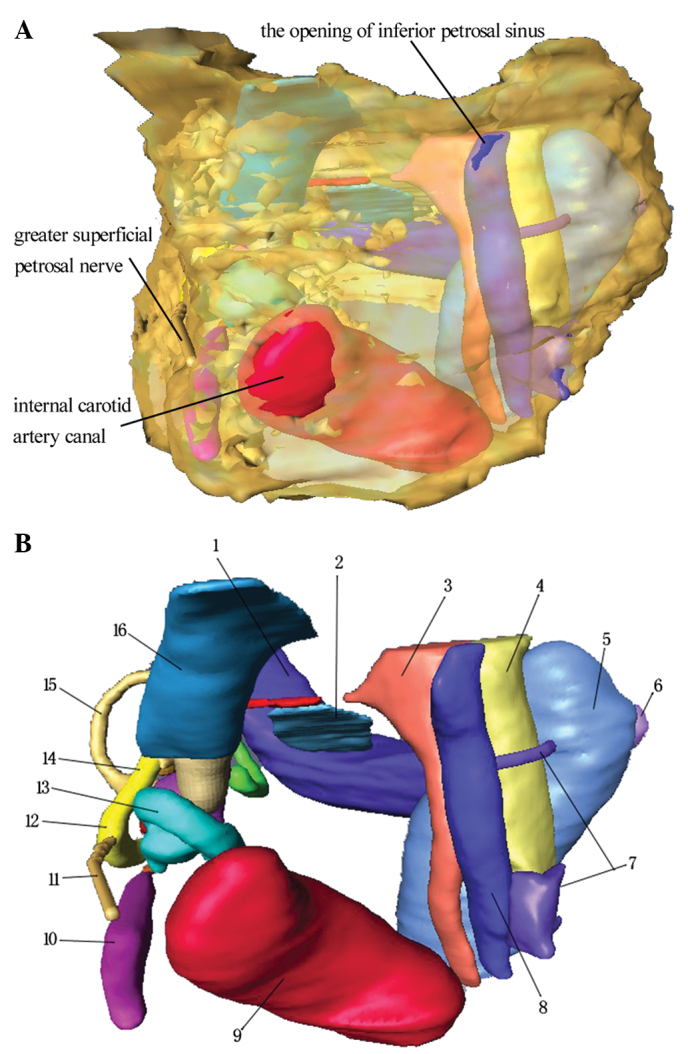
Anteromedial view of the petrous bone model. (A) Semi-transparent view of the petrous bone. (B) Following complete transparency of the petrous bone. 1, sigmoid sinus; 2, extraosseous portion of the endolymphatic sac; 3, glossopharyngeal meatus; 4, vagal meatus; 5, internal jugular vein canal; 6, condylar canal; 7, branch of the IPS; 8, IPS; 9, internal carotid artery canal; 10, tensor tympanic muscle; 11, greater superficial petrosal nerve; 12, geniculate ganglion; 13, cochlea; 14, facial nerve canal; 15, superior semicircular canal; 16, internal acoustic meatus. IPS, inferior petrosal sinus.

**Figure 5:**
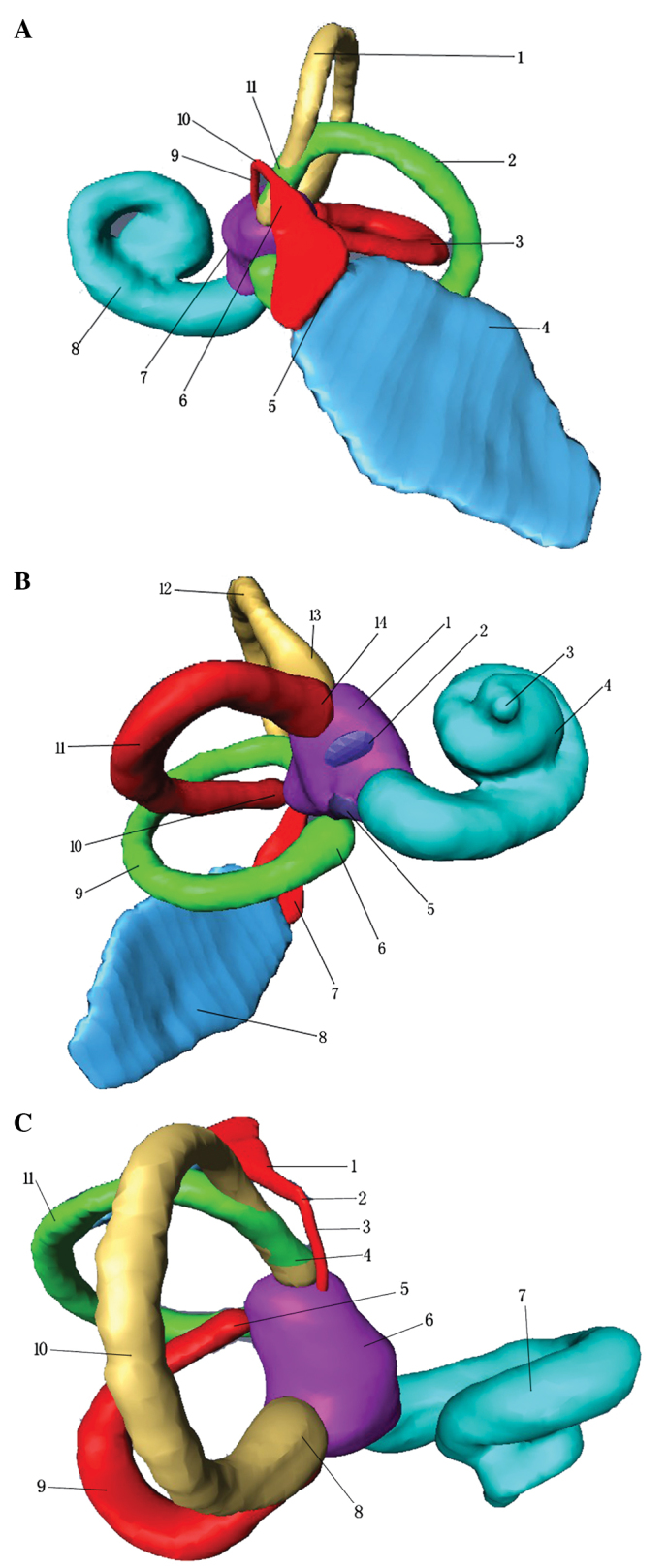
Association among the VA, ES and bony labyrinth. (A) Inferior view. 1, superior semicircular canal; 2, posterior semicircular canal; 3, lateral semicircular canal; 4, extraosseous portion of the ES; 5, external aperture of the VA; 6, distal VA; 7, vestibule; 8, cochlea; 9, proximal VA; 10, isthmus of the VA; 11, crus commune. (B) Anterolateral view. 1, vestibule; 2, oral window; 3, apex of the cochlea; 4, cochlea; 5, round window; 6, ampulla of the posterior semicircular canal; 7, VA; 8, extraosseous portion of the ES; 9, posterior semicircular canal; 10, crus simplex; 11, lateral semicircular canal; 12, superior semicircular canal; 13, ampulla of the superior semicircular canal; 14, ampulla of the lateral semicircular canal. (C) Superior view. 1, distal VA; 2, isthmus of the VA; 3, proximal VA; 4, crus commune; 5, crus simplex; 6, vestibule; 7, cochlea; 8, ampulla of the superior semicircular canal; 9, lateral semicircular canal; 10, superior semicircular canal; 11, posterior semicircular canal. VA, vestibular aqueduct; ES, endolymphatic sac.

The petrous bone with its surface landmarkers could be observed posteriorly, anteromedially (Fig. 4) or inferiorly through the 3D model. All reconstructed structures could be represented individually or jointly and rotated continuously in various planes. The mutual positional relationship among the three semicircular canals, cochlea, vestibule, round window, oval window, VA and ES is shown in Fig. 5. The three semicircular canals communicate with the vestibule via five openings, one of which is formed by the union of the non-ampullated ends of the superior and posterior canals, known as the ‘common crus’. The spiral cochlea began at the vestibule and made two-and-a-half turns around the cone-shaped modiolus for distribution of the cochlear nerve. It lay between the internal auditory canal and the tympanic cavity, inferiorly associated with the jugular vein. The VA coursed upward from the vestibule, bent near the isthmus portion, and then coursed downward and widened on approach to the external aperture. The ES extended through the distal VA and out the external aperture of the aqueduct to terminate in the epidural space of the posterior cranial fossa.

### Association between the ICA canan, IJV canal, SS, IPS, glossopharyngeal meatus and vagal meatus

When the ICA entered the canal in the petrous portion of the temporal bone, it first ascended a short distance and then curved forward and medially. The ICA can be observed adjacent to the basal turn of the cochlea (Fig. 4B). The Jugular foramen (JF) is an important bony channel of the posterior fossa. The dura overlying the intrajugular compartment formed two perforations. One of these perforations was the glossopharyngeal meatus, through which the glossopharyngeal nerve passes; the other was the vagal meatus, through which the vagus and accessory nerves pass. The SS is the largest source of venous drainage into the JF (10). The IPS formed a multi-channel confluence that emptied into the IJV between the glossopharyngeal and vagus nerves in 1–3 roots. The internal opening of the condylar canal was located at the posteromedial margin of the IJV.

### Association between the IAM and facial nerve canal

The internal auditory meatus is a canal in the petrous bone that carries nerves, i.e. cranial nerves VII and VIII, from inside the skull towards the inner ear. Near the bottom of the IAM, openings exist for three different canals (Fig. 4). The anterosuperior canal transmits the facial nerve and is separated from the posterosuperior section, which transmits the superior vestibular nerve, by Bill’s bar. The cochlear nerve runs antero-inferiorly and the inferior vestibular nerve runs postero-inferiorly. The facial canal is a Z-shaped canal running through the temporal bone from the fundus of the IAM to the stylomastoid foramen. The greater superficial petrosal nerve can be observed extending anteriorly over the basal turn of the cochlea and the ICA. The tensor tympani muscle was observed to turn laterally along the cochleariform process, which was located at the anterior part of the horizontal segment of the facial nerve.

## Discussion

The process of learning the surgical anatomy of the petrous bone is challenging due to the small but complex structure of the bone. The rapid development of computer technology has led to the potential for its use in modeling and simulating the human anatomy. The first computer-generated 3D model of the temporal bone was produced by Antunez *et al* in 1980 (11). To create this model, images were captured of each histological section and the images were then transferred to a computer graphics tablet. From this, the computer generated surface-rendered 3D models of the ES and the endolymphatic duct. In 1989, Lutz *et al* (12) examined 60 sagittal histologic sections of a normal left temporal bone, and data were entered into a computer for the 3D reconstruction of a temporal bone. Over the past two decades, there have been marked improvements in temporal bone reconstruction. In 2001, Zieliński and Słoniewski (1) created a virtual, 3D computer model of the petrous bone based on 1-mm tomographic X-ray slices. One year later Page *et al* (13) highlighted the feasibility of creating 3D images of the petrous bone from a routine CT examination. Bernardo *et al* (14) designed a 3D surgical simulator known as interactive virtual dissection, which allowed the user to drill progressively deeper into the petrous bone and to identify crucial structures. Tang *et al* (15) evaluated the application of a virtual reality system in the construction of 3D petrous bone. The Digital Imaging and Communications in Medicine data of the CT performed for 15 adult cadaver heads were transferred to and reconstructed in the Destroscope virtual reality system. The use of 3D imaging enabled the illustration of the distinct spatial relationship of anatomic structures associated with the petrous bone. A limitation of these previous studies, however, was that they showed only a few of the internal structures of the petrous bone and their mutual relationships. There remains a lack of a 3D model that can be used to observe all the structures of the petrous bone, and to make an association between superficial and deep structures.

In this study, a 3D model of the petrous bone, which systematically displayed the detailed structures of the bone (including soft tissue and spatial locations), was generated. This is likely to aid the understanding of otologists with regard to the spatial relationships of temporal bone structures. Transparency allowed the visualization of substructures such as the bony labyrinth ‘inside’ the hard petrous pyramid. All reconstructed structures could be represented individually or jointly and rotated continuously in any plane (Fig. 5). Since the reconstructed images of the intratemporal bone can be viewed from all surgical angles and numerous spatial relationships can be accurately observed, the model may provide a guide for otosurgery and enhance medical education. Previously, focus was placed on the generation of 3D models for a structure or part of a structure inside the petrous bone. Li *et al* (2), for example, sliced the right ear of a male cadaver (14 years old) with celloidin, and then modeled the bone structures with Amira software and studied the connection between the structures. There is, however, a lack of comprehensive description based on a 3D model depicting the inside structures of the petrous bone, including the IJV canal, carotid artery, glossopharyngeal channel, three semicircular canals and internal auditory canal, which has largely limited the anatomical understanding of this region. On this basis, the present study has provided a unique comprehensive presentation of the 3D structure inside the petrous bone, and thus may enhance the understanding of the intrinsic connection between different parts of the bone.

The methods of acquiring digital information from mammals include micro-CT, MRM and orthogonal-plane fluorescence optical sectioning microscopy (OPFOS) (16). Previous studies have predominantly adopted technologies such as CT and MRI, which have resulted in thick slices (10 and 86 μm, respectively) at low resolution, as well as the loss of structural information due to the gray scaling strategy (9,17–24). Consequently, this has impaired the accuracy of reconstruction for the fine structures of the inner ear. Although comparatively high image resolution can be obtained with OPFOS, there is a decline in image quality due to the dimensions of the human inner ear, which make it difficult to describe the precise configuration in the petrous bone. Images of celloidin sections are therefore the best 2D digital material for reference. In the present study, 40-μm, serial, unstained collodion slices of the petrous bone, which showed the inner ear, were utilized for the reconstruction of 3D models on this basis. These efforts largely avoided the loss of refined structural information about the inner ear, and ensured the consistency in morphology between the modeled VA and the real structure. Furthermore, technologies such as CT and MRI are largely limited to partial structures inside petrous bone, while certain other regions, such as the ES, glossopharyngeal channel and the bottom of internal auditory canal, can only be observed and thus modeled on the basis of the collodion films.

In previous studies, the sampling of every fifth to 10th section stained has typically been applied, producing considerable error (25,26). As described by Rother *et al* (25), when specimens were prepared in the typical manner at 20 μm, and only every fifth to 10th section was scanned, the obtained measurements had an error of ±0.1–0.2 mm in a strictly vertical plane. The use of every fifth to 10th section for 3D reconstruction thus results in the loss of considerable amounts of vital information regarding these tiny structures, which evidently affects the precision of the 3D model. In the present study, all the intact sections were employed and any missing sections were replaced with adjacent ones. Additionally, in the segmentation procedure, the boundaries of the sections that replaced the missing ones were moved to the middle of the two adjacent ones, thereby further reducing the error.

A process with fewer steps is likely to lead to a smaller error margin. A complicated staining procedure could damage some of the celloidin sections, and unpredictable distortion could occur during dehydration, clearing or the other procedures (26). In the present study, any potential causes of error were avoided by using unstained sections, which also reduced laboratory workload to a great extent. Furthermore, in the images of the unstained sections, the small structures of the petrous bone were distinct enough for precise segmentation, as described earlier in the study (Fig. 1).

It is worth noting that, despite the significant advantages of our comprehensive model of the complete petrous bone structure, the model also exhibits limitations, such as poor resolution and potential error in the sampling procedure; however, these limitations do not affect the essential findings of the study. Efforts will be made to improve the quality and reliability.
